# Presence and Function of Dopamine Transporter (DAT) in Stallion Sperm: Dopamine Modulates Sperm Motility and Acrosomal Integrity

**DOI:** 10.1371/journal.pone.0112834

**Published:** 2014-11-17

**Authors:** Javier A. Urra, Franz Villaroel-Espíndola, Alejandra A. Covarrubias, Joan Enric Rodríguez-Gil, Alfredo Ramírez-Reveco, Ilona I. Concha

**Affiliations:** 1 Escuela de Graduados, Facultad de Ciencias Veterinarias, Universidad Austral de Chile, Valdivia, Chile; 2 Instituto de Bioquímica y Microbiología, Facultad de Ciencias, Universidad Austral de Chile, Valdivia, Chile; 3 Instituto de Ciencia Animal, Facultad de Ciencias Veterinarias, Universidad Austral de Chile, Valdivia, Chile; 4 Unitat de Reproducció Animal, Facultat de Veterinària, Universitat Autònoma de Barcelona, Bellaterra, Barcelona, Spain; Universidad Nacional Autónoma de México, Mexico

## Abstract

Dopamine is a catecholamine with multiple physiological functions, playing a key role in nervous system; however its participation in reproductive processes and sperm physiology is controversial. High dopamine concentrations have been reported in different portions of the feminine and masculine reproductive tract, although the role fulfilled by this catecholamine in reproductive physiology is as yet unknown. We have previously shown that dopamine type 2 receptor is functional in boar sperm, suggesting that dopamine acts as a physiological modulator of sperm viability, capacitation and motility. In the present study, using immunodetection methods, we revealed the presence of several proteins important for the dopamine uptake and signalling in mammalian sperm, specifically monoamine transporters as dopamine (DAT), serotonin (SERT) and norepinephrine (NET) transporters in equine sperm. We also demonstrated for the first time in equine sperm a functional dopamine transporter using *4*-[*4*-(*Dimethylamino*)styryl]-N-methylpyridinium iodide (ASP^+^), as substrate. In addition, we also showed that dopamine (1 mM) treatment *in vitro*, does not affect sperm viability but decreases total and progressive sperm motility. This effect is reversed by blocking the dopamine transporter with the selective inhibitor vanoxerine (GBR12909) and non-selective inhibitors of dopamine reuptake such as nomifensine and bupropion. The effect of dopamine in sperm physiology was evaluated and we demonstrated that acrosome integrity and thyrosine phosphorylation in equine sperm is significantly reduced at high concentrations of this catecholamine. In summary, our results revealed the presence of monoamine transporter DAT, NET and SERT in equine sperm, and that the dopamine uptake by DAT can regulate sperm function, specifically acrosomal integrity and sperm motility.

## Introduction

Dopamine is a catecholamine that participates in many biological processes in mammals, acting on functions related to cognition, emotions and control of motor activity, among others [1]. In the reproductive system, high concentrations of catecholamines have been observed in bovine [2] and human [3] semen as well as in the oviduct of human, pig, rabbit and cow [4], [5], [6], [7] at variable concentrations dependent on the oviductal region and the estrous cycle stage analyzed [5], [6], [7]. It is presumed that these catecholamines come from sympathetic nerve endings that innervate both testis and oviduct. With respect to this, there is evidence of expression of tyrosine hydroxylase (TH), a rate-limiting enzyme in catecholamine synthesis, in the uterus and cervical cells of mare [8], in neuronal-type cells of non-human primates [9] and in human Leydig cells [10]. This raises the possibility that catecholamines are synthesized from a source other than that of the innervation present in testis and oviduct, implying that sperm would be in contact with catecholamines, or at least with L-DOPA, a precursor of dopamine, from a very early stage during their passage through the male and female reproductive tract.

Evidence shows that catecholamines exert their actions on different parameters of sperm physiology. They have been shown to induce capacitation in mouse [11], hamster [12], [13] and bull sperm [14], as well as stimulating motility *in vitro*, in hamster sperm [15] and promoting the acrosome reaction in hamster [16], [13] and bovine sperm [14].

Presence of different catecholaminergic receptors in sperm has been reported, including the presence of α2- and β-adrenergic receptors in mouse and human sperm [11], [17] and the dopamine type 2 receptor (DRD2) in testis, spermatogenic cells and sperm of rat, as well as in human, bull, mouse and boar sperm [18], [19]. Previously, we reported that boar sperm incubated in the presence of 100 nM dopamine displayed a significant decrease in the number of dead sperm at different incubation times (up to 3 hours) compared to the control, also this treatment promoted a significant increase in proteins phosphorylated on tyrosine in sperm incubated with dopamine. These results suggest a protective effect of dopamine treatment on sperm and dopamine would be acting as a modulating agent in sperm capacitation [19]. However, when boar sperm were incubated with higher concentration of dopamine (1 mM), a decrease in both protein-tryosine phosphorylation and sperm motility was observed [19]. Similar findings have been reported for norepinephrine in bull sperm, where incubation with 3.1 µM norepinephrine stimulates an increased phosphorylation of proteins on tyrosine residues and the acrosome reaction, whereas incubation with 3.1 mM norepinephrine resulted in both parameters being negatively affected [14]. This biphasic effect of catecholamines concentrations might be explained by the presence of adrenergic receptors and DRD2 in those findings observed at low doses and by the possible presence of catecholamine transporters for findings observed at higher doses of catecholamines. Western blot and immunocytochemical analyses in equine sperm suggest the presence of the dopamine transporter, DAT, however a kinetic analysis and functional characterization are absent [19].

In the present study we show the presence of DAT transporter in equine sperm and we investigate this transporter function and its participation in equine sperm function. We also demonstrate the presence of the SERT and NET transporters in equine sperm. These results suggest a high degree of conservation of the mammalian catecholaminergic system and the participation of dopamine in the control of the overall mammalian sperm function.

## Results

### Functional dopamine transporter in mammalian sperm

**Figure 1:**
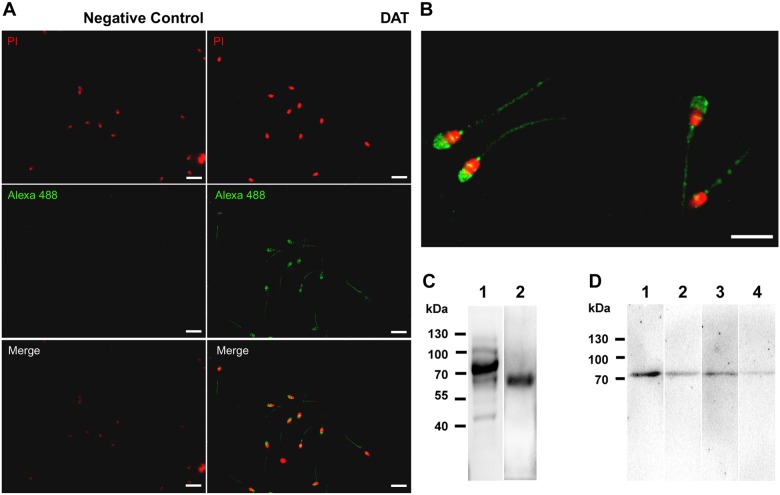
Dopamine transporter (DAT) is present in equine ejaculated sperm. The presence of DAT in ejaculated equine sperm was investigated with immunodetection and microscopy methods. **A)** Sperm were fixed and analyzed by immunofluorescence using an anti-DAT antibody or only secondary antibody as negative control. **B)** Magnification of DAT immunodetection in sperm. Bar scale is 10 µm. **C)** 80 µg of total protein extract from rat brain (line 1) and equine sperm (line 2) were analyzed with SDS-PAGE and Western blot using a specific human anti-DAT antibody. Images are representative of 3 independent experiments. **D)** UREA/SDS-PAGE and Western blot analysis for total protein extract (100 µg) for rat brain (line 1 and 3) and equine sperm protein extract (line 2 and 4), with (line 2 and 4) or without (Line 1 and 3) anti-Dat antibody preabsorbed by immunogenic peptide (SC-7515 P).

In boar sperm, incubation with dopamine alters different parameters of sperm physiology in a dose-dependent manner [19]. Here, we found in equine sperm, similar to boar sperm, that high concentrations of dopamine (1 mM) decreased sperm motility, tyrosine phosphorylation and acrosome integrity in *in vitro* sperm capacitation assays. Using immunofluorescence and Western blot assays, we have demonstrated the presence of the dopamine transporter in equine sperm (Figure 1) and other species (Figure S1). For equine sperm, a strong immunoreaction was detected in the acrosomal region and less immunoreactivity was observed in the principal piece of the sperm tail (Figure 1A and 1B). Negative results were obtained when sperm samples were incubated only with secondary antibody (Figure 1A). Immunoblotting against DAT in extracts from both brain (as control) and sperm showed the presence of an unique, specific protein band of 70 kDa in whole protein extract from equine sperm, and several bands (possibly different degrees of glycosylation) in the positive control extracts (between 45–120 kDa), with a major band of 75 kDa (Figure 1C), confirming the presence of this transporter in these cells. Figure 1 D corresponds to Western blot analysis of both the control and sperm protein samples (lines 1 and 3, respectively for DAT immunodetection). The immunoreaction largely diminished by using the preabsorbed antibody with immunogenic peptide (Figure 1D, lines 2 and 4, respectively). Urea treatment promoted the immunodetection of a unique band in both samples (Figure 1D), confirming the specifity of this result.

**Figure 2:**
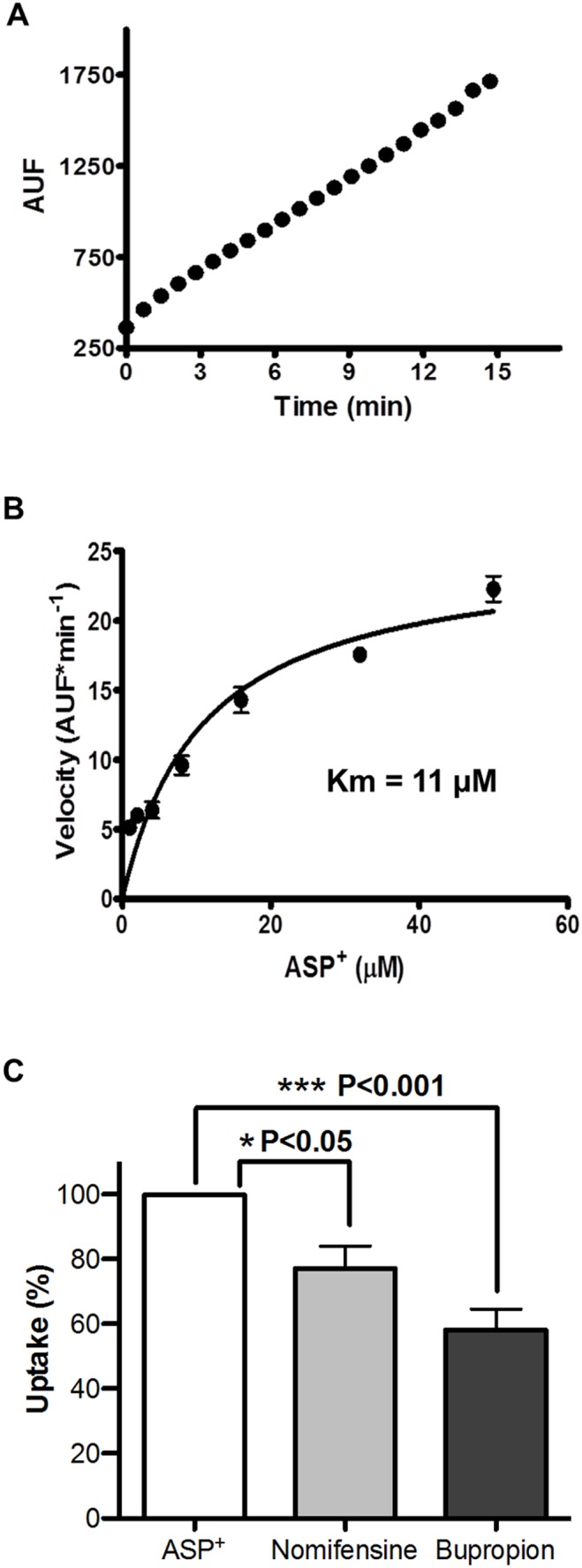
The dopamine transporter present in equine sperm is functional and sensitive to selective inhibitors. **A)** In freshly ejaculated equine sperm, ASP^+^ transport was used to assess the functionality of the DAT transporter. Fresh sperm were incubated with 8 µM ASP^+^ in capacitation medium and were analyzed with fluorimetry. Accumulated fluorescence was plotted as arbitrary units of fluorescence (AUF) as a function of transport time. Measurements were made every 35 seconds over a total assay time of 15 minutes. Results were plotted as the mean ± standard error (SEM) of eight independent assays. **B)** Kinetic characteristics of the DAT transporter present in equine sperm were established by incorporation of ASP^+^. Fresh sperm were incubated with different ASP^+^ concentrations (0 to 30 µM) in capacitation medium for 20 minutes. The difference between fluorescence at time zero and at twenty minutes was plotted as velocity with respect to the different ASP^+^ concentrations used in the assay. Results correspond to the mean ± standard error of an average of eight independent experiments. **C)** DAT transporter sensitivity was assessed in response to selective inhibition with 50 µM nomifensine and 10 µM bupropion. Fresh sperm were incubated with 8 µM ASP^+^ in capacitation medium for 20 minutes and the difference between fluorescence at time zero and at twenty minutes was recorded. Transport of ASP^+^ was considered to be 100% and inhibitor treatments were normalized with respect to this. Results correspond to the mean ± standard error (SEM) of an average of eight independent experiments, *p<0.05.

We also evaluated the function of the DAT transporter in equine sperm. In fresh sperm preparations, ASP^+^ incorporation assays show a linear accumulation of the fluorescent molecule during the 15 min of the assay (Figure 2A). Saturation analysis of the DAT transporter gives a Km value of 11 µM (Figure 2B). The inhibition assays using 50 µM nomifensine (Ki 2.6 µM for dopamine uptake) and 10 µM bupropion (Ki 2.8 µM for dopamine uptake) revealed a significant reduction in ASP^+^ uptake and accumulation in equine sperm. Both nomifensine and bupropion reduced dopamine analog (ASP+) transport by 24±6% and 42±6% respectively, values that are significantly lower with respect to the control without inhibitor treatment (Figure 2C).

### High doses of dopamine reduce sperm motility, acrosomal integrity and tyrosine phosphorylation in equine sperm

**Figure 3:**
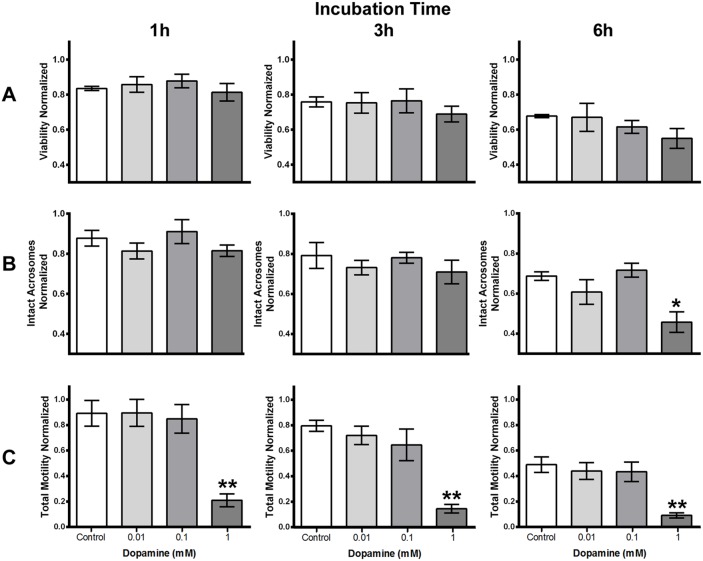
High doses of dopamine reduce sperm motility and acrosomal integrity without affecting the sperm viability over time. **A)** Effect of dopamine on sperm viability at 1, 3 and 6 hours of incubation. Equine sperm were incubated at 37°C with different concentrations of dopamine (0, 0.01, 0.1 and 1 mM) in Withenn’s buffer and the quantity of viability sperm was calculated using a CASA system, with a minimum of 500 sperm analyzed per experiment. Sperm viability at different times of analysis was normalized to time zero of the control. Results are the mean ± SEM of four independent experiments. **B)** Effect of dopamine on acrosomal integrity after 1, 3 and 6 hours of incubation. Equine sperm were incubated at 37°C with different dopamine concentrations (0, 0.01, 0.1 and 1 mM) in Withenn’s buffer (enrichment with BSA and bicarbonate) and the percentage of sperms with intact acrosomes was assessed with PSA-FITC staining and by cell count under an epifluorescence microscope. A minimum of 200 sperm were counted. The percentage of intact acrosomes was normalized to time zero of the control without dopamine. Results are the mean ± SEM of four independent experiments. *p<0.05 with respect to the control. **C)** Effect of dopamine on total sperm motility after incubation for 1, 3 and 6 hours. Equine sperm were incubated at 37°C with different concentrations of dopamine (0, 0.01, 0.1 and 1 mM) in Withenn’s buffer and the quantity of mobile sperm was calculated using a CASA system. A minimum of 500 sperm were analyzed per experiment. Total motility at different times of analysis was normalized to time zero of the control. Results are the mean ± SEM of four independent experiments. **p<0.01 with respect to the control.

After having established the presence of a dopamine transporter at the sperm surface, the effect of high concentrations of dopamine (0.01, 0.1 and 1 mM) on sperm cell viability, acrosomal integrity and sperm motility were evaluated at three incubation times. The results obtained at 1, 3 and 6 hours did not reveal significant changes in sperm viability at the different concentrations of dopamine used (Figure 3A). The effect of high dopamine doses on acrosomal integrity of ejaculated sperm was also investigated. Sperm exposed to increasing doses of dopamine (0.01, 0.1 and 1 mM) for 1 or 3 hours, showed no significant changes in acrosomal integrity (Figure 3B). Results obtained after 6 hours of exposure show that only concentrations as high as 1 mM dopamine provoked a significant decrease in sperm acrosome integrity, which is reflected by an almost 35% reduction of intact acrosomes with respect to the control without treatment (Figure 3B).

**Figure 4:**
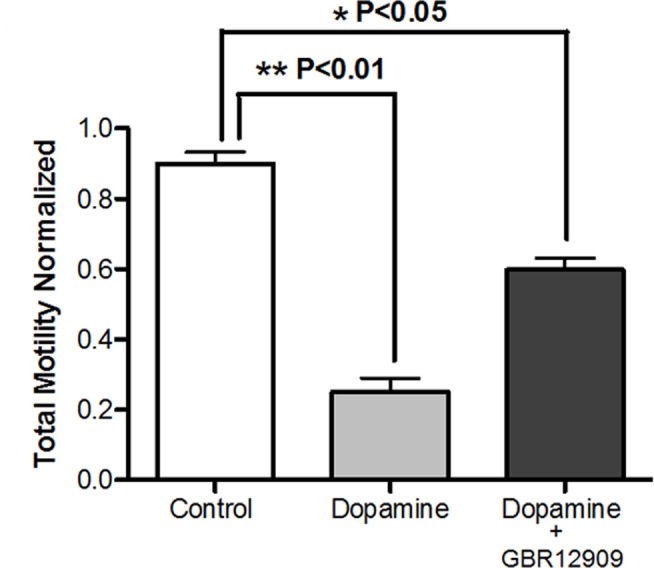
Dopamine transporter inactivation attenuates the inhibitory effect of 1 mM dopamine on total sperm motility. Equine sperm were incubated at 37°C with 1 mM dopamine, with and without the specific DAT inhibitor, 10 µM GBR12909 for 1 hour, in Withenn’s buffer. Total motility was calculated using the CASA system, with a minimum of 500 sperm analyzed per experiment. Total motility was normalized to time zero of the control. Results are the mean ± SEM of four independent experiments. The different letters show significant changes between each treatment with p<0.01. We have considered in each experiment a control to normalize the results to the basal sperm condition before the incubation with dopamine and its respective vehicle.

Total sperm motility in the presence of 0.01, 0.1 and 1 mM dopamine was also determined. We observed a strong inhibition in total sperm motility only at dopamine concentration of 1 mM after incubating for 1, 3 and 6 hours (Figure 3C), similar response was observed in progressive sperm motility analysis (data non shown). Complementarily, fresh sperm were exposed to 1 mM dopamine in the absence and presence of 10 µM GBR12909, a strong and specific DAT inhibitor (Ki 1 nM for dopamine uptake). We found that the dopamine-induced inhibition of total sperm motility (75%) was partially reversed (40%) in the presence of 10 µM GBR12909 (Figure 4).

**Figure 5:**
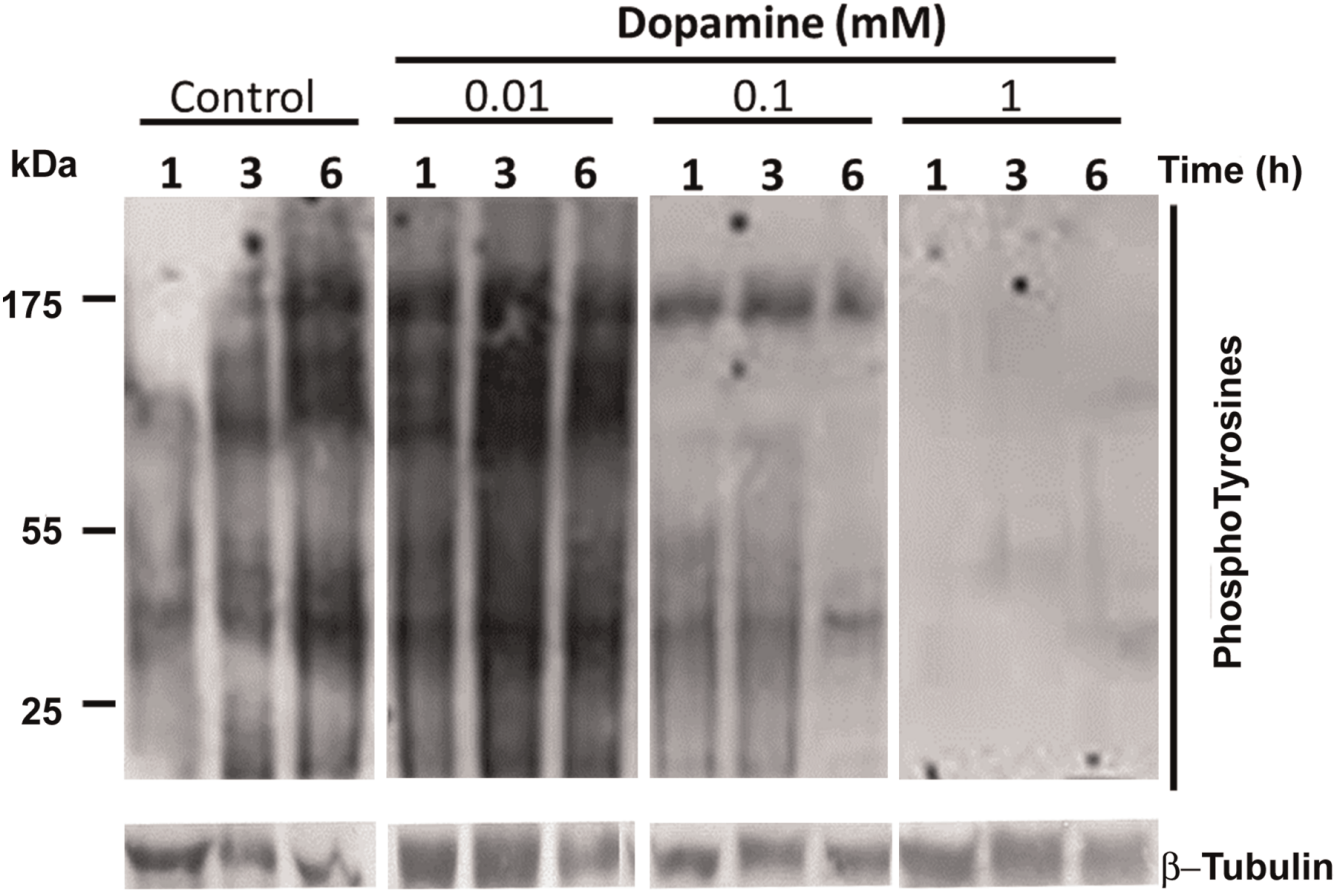
High levels of dopamine reduce tyrosine phosphorylation during capacitation in equine sperm. Effects of dopamine on tyrosine phosphorylation after different times of incubation and dopamine concentrations (0, 0.01, 0.1 and 1 mM) were assessed by Western blot using PY20 anti-phophotyrosine antibody. The image is representative of two independent experiments.

Finally, to evaluate possible signalling pathways involved in the negative regulation of sperm motility by high doses of dopamine, we assessed the overall pattern of tyrosine phosphorylation. Major changes were observed on tyrosine phosphorylation during different incubation times, with a biphasic and dose dependent behavior, increassing their level at 10 µM dopamine and decreassing their level with dopamine at 100 µM and 1 mM (Figure 5).

### Norepinephrine and serotonin transporters are expressed in equine sperm

**Figure 6:**
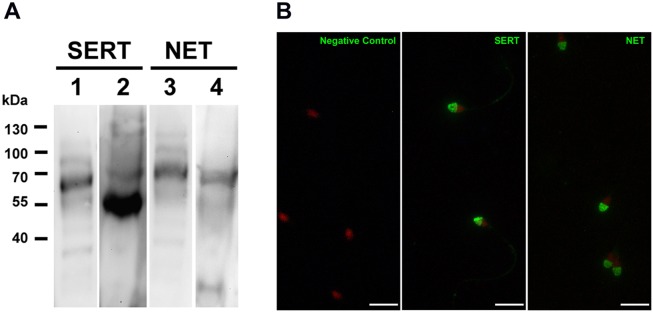
SERT and NET are present in ejaculated sperm. The presence and location of other monoamine transporters was determined in equine sperm. A) The presence of SERT and NET transporters were verified in equine sperm (line 2 and 4) by Western blot assays. A protein extract from whole rat brain (line 1 and 3) was used as a control. Images are representative of 3 independent experiments. B) The localization of SERT and NET was performed by indirect immunofluorescence assays. Images are representative of 3 independent experiments. Bar scale is 10 µm.

Using Western blot and fluorescent immucytochemical analyses, we evaluated the presence of other monoamine transporters that may be involved, albeit with lower selectivity in the incorporation of dopamine in equine sperm. The presence of NET and SERT transporters in equine sperm was verified by immunodetection in whole protein extract (Figure 6A). NET and SERT were detected in equine sperm in form of specific bands of 70 kDa and 58 kDa, respectively (Figure 6A).

By immunofluorescence assay, NET distribution was specific in acrosome region in the head sperm (Figure 6B). A similar pattern was found for SERT in the head, which also presented a weak signal in the mid-piece and the tail (Figure 6B).

## Discussion

Our results clearly indicate that equine sperm (and other mammalian species) express a wide range of proteins from the dopaminergic system, specifically monoamine transporters, such as dopamine, norepinephrine and serotonin transporters. These findings, together with existing knowledge from other research literature [18], [19] reveals the presence of a dopaminergic-type system in sperm cells, suggesting that sperm cells contains several proteins for binding and uptake of catecholamines as signaling molecules or modulators of sperm physiology in mammals.

Mammalian sperm are exposed to variable concentrations of catecholamines during their passage through the reproductive tracts, both male and female. Catecholamines, especially norepinephine, have been reported to participate in testicular function [20], [21], in sperm travel from the epididymis through the vas deferens [22], [23]. Other studies performed in different portions of human fallopian tubes (isthmus, ampulla and fimbriae) showed that the highest concentrations of catecholamines are found during the preovulatory and ovulatory phases [5]. Considering the effect of high doses of dopamine in *in vitro* sperm function, particularly in the control of sperm motility, maintenance of acrosomal integrity and tyrosine phosphorylation in a wide range of target proteins, it might be suggested that these higher catecholamine concentrations could participate in the modulation of *in vivo* sperm function. This hypothesis is reinforced by previous results from our laboratory in which both total and progressive motility and also phosphotyrosines of boar sperm in *in vitro* capacitation assays were inhibited by dopamine without affecting sperm cell viability [19]. On the other hand, lower concentrations of catecholamines have been found in the ampullary region, where fertilization takes place, as in the post-ovulation phase, in which sperm motility is recovered and sperm are released from the reservoir for fertilization [5]. It is also in concordance with our previous results in boar sperm, which showed that low concentrations of dopamine (100 nM) potentiate sperm motility, and total phosphotyrosines [19], suggesting a putative stimulatory effect of dopamine on sperm motility in this region of the female reproductive tract.

We have hypothesized that catecholamine could be concentrated in micro-domains from mammalian oviduct (ampulla and isthmus region) and promote a sperm selection during ovulation. Many authors have reported higher concentration of catecholamine in oviductal fluid at different stages of ovarian activity. During rabbit’s oestrus it has been found that dopamine and norepinephrine concentrations are close to 8 and 27 µM, respectively in the isthmus region, and oscillating dopamine concentrations between 1–10 µM during ovulation and coitus [6]. Serum concentrations of dopamine and norepinephrine in rabbit are close to 600 nM and 5 nM, respectively [24], [25]. On other hand, the physiological concentration of dopamine measured in equine plasma are close to 3.5 µM, and norepinephrine and epinephrine are 0.3 and 0.1 µM, respectively [26]. Up today, there is no information about dopamine levels in equine seminal or oviductal fluid. However, plasma concentrations of dopamine outlined above are 5 times higher than in other mammals, suggesting that these would also be more elevated in both semen and oviductal fluid.

The ASP^+^ transport assays demonstrate the presence of a functional dopamine transporter in equine sperm, suggesting that this transporter would be capable of receiving and generating the modulatory effects of this catecholamine. The Km obtained for this transporter was of approximately 11 µM. There seem to be no previous measurements of equine DAT transport activity in the literature, but the Km of DAT for ASP^+^ in human, using cultured HEK293 cells transfected with human DAT, reveal a Km of 3.2 µM [27], [28]. We have demonstrated that DAT transporter has a Km 3 times higher than human (and mouse), suggesting that this transporter has lower affinity for dopamine and could require higher levels of substrate to operate. This higher value of Km suggests an insensitivity of DAT transporter to lower doses of this catecholamine, which was demonstrated in this work and on the other hand, higher doses would have deleterious effects on sperm physiology.

However, we cannot dismiss the participation of the NET and SERT transporters in the uptake of ASP^+^ [29], [30], given that we have verified and demonstrated the presence of both transporters in ejaculated equine sperm. This situation would explain the partial reversal observed with 50 µM nomifensine and 10 µM bupropion on the in ASP^+^ uptake (Figure 2C).

An unexpected event is that 50 µM nomifensine completely fails to inhibit the incorporation of ASP^+^ by DAT, NET and SERT (2.6 µM, 4.7 µM and 4 nM Ki for the incorporation of dopamine, respectively), on the other hand, 10 µM bupropion would be sufficient to completely inactivate DAT and NET, but not SERT (2.8 µM, 1.4 µM and 45 µM of Ki for the incorporation of dopamine, respectively). We have not disregarded the presence of non-classical monoamine transporters or non-neuronal transporters that are not affected by classical inhibitors of monoamine transporters [31], suggesting thus that the modulatory role of dopamine on sperm physiology may be a complex, as yet unknown panorama.

In summary, we have demonstrated that equine sperm express dopamine receptors and transporters. Moreover, our results indicate that this dopaminergic system is functional in the studied sperm. Considering that catecholamines are present throughout the entire passage of sperm along the male and female reproductive tracts, it is logical to think that they may fulfill some function in sperm and reproductive physiology. We have also shown that high dopamine concentrations dramatically affect sperm motility without altering viability; however these sperm could experiment a decrease in its hability to capacitation. In light of the results obtained in this study, greater concentrations of dopamine could help immobilize sperm in the oviductal reservoirs promoting a possible positive selection of the best sperm until the ovulation, just at the moment in which catecholamine levels fall. Subsequently, other factors present in oviduct and follicular fluid could stimulate sperm motility and capacitation, thus enhancing the oocyte penetration in the appropriate segment. Finally, the gradient in the concentration of dopamine in the oviduct (and oviductal/follicular fluids) would be a simple and effective system to modulate the sperm physiology in the oviduct and optimizing the fertility in equine.

## Materials

Antibodies anti DAT (sc-7515), NET (sc-51157), SERT (sc-1458), DAT peptide (sc-7515 P), nomifensine (sc-253197) and bupropion (sc-217802) were obtained from Santa Cruz Biotechnology, Inc. Anti PY20 (P4110) and anti β-tubulin (T5201), dopamine (H8502), tetrazolium blue (N5514) and PSA-FITC (L0770) were obtained from Sigma Aldrich. ASP^+^ (D289), anti-goat Alexa 488 (A11055) were obtained from Invitrogen and vanoxerine (GBR12909–[0421]) was obtained from Tocris Bioscience. Other reagents used were acquired from Sigma Aldrich or Merck.

## Methods

### Ethics statement

Human semen samples were obtained by masturbation and collected in sterile plastic containers from volunteer’s healthy young men and with previous written consent, the protocols used in this study were approved by the Committee Bioethic of Medical Science of Universidad Austral de Chile (CI#213-2014).

The stallions samples used were obtained from animals following the regulations of the Committee on the Bioethics of Animals for Research of Universidad Austral de Chile, instance that reviewed and approved the protocols used in this study (C#151-2014). The only manipulation of animals was semen collection using standard procedures established by Haras Militar Pupunahue (Ejército de Chile). Bovine sperm samples used correspond to frozen commercial doses obtained from Centro de Inseminación Artificial (Universidad Austral de Chile). Boar sperm samples used correspond to refrigerated commercial doses obtained from Servicios Genéticos Porcinos, S. L. (Roda de Ter, Spain).

### Sperm samples

#### Equine sperm

Ejaculates were obtained from six stallions (Heavy Draft breeds): Ardennés (2), Percherón (2) and Bretón de Montaña (2), aged between 7 and 15 years, property of Haras Militar Pupunahue, XIV Región de los Ríos, Máfil, Valdivia, Chile. The Haras Militar Pupunahue, as a partner member of the Fondef D08I1076 proyect, explicitly approved the use of the samples for scientific research purposes. All stallions were proven to be fertile. Ejaculated sperm obtained and used for the study had viability and motility parameters of greater than 70 and 65% respectively.

Semen was collected with the aid of an artificial vagina, was filtered to eliminate the gel fraction and was diluted to a ratio of 1∶1 with UHT skimmed milk at 37°C and refrigerated at 4°C for transport to Laboratory of Cryobiology and Sperm Function Analysis, Universidad Austral de Chile, Valdivia. Once in the laboratory, semen was centrifuged at 600×g for 20 minutes and the pellet re-suspended in incubation medium or Whitten’s medium (100 mM NaCl, 4.7 mM KCl, 1.2 mM MgCl_2_, 5.5 mM glucose, 22 mM Hepes, 4.8 mM lactic acid hemicalcium salt, 1 mM pyruvate, pH 7.25) in the absence of albumin (BSA), sodium bicarbonate and progesterone. Samples were maintained at 4°C until required for different assays. Previous to each experimental procedure, sperm viability and motility was measured, rejecting those ejaculated sperm with a viability of less than 70% and a motility of less than 65%. Whitten’s medium modified (enrichment with BSA and bicarbonate) was used during each assay performance, and all experiments were done at different times of incubation (in presence or absence of dopamine) by 1, 3 or 6 hours. In each case, as control, was evaluated the basal status of viability, acrosomal reaction or motility, previous to the start experiment and when the experiment was finished.

#### Bovine, boar and human sperm samples for Western blot analisys

Bovine spermatozoa ejaculates were obtained from the Centro de Inseminación Artificial Universidad Austral de Chile. Boar sperm samples used correspond to refrigerated commercial doses obtained from Servicios Genéticos Porcinos, S.L.(Roda de Ter,Spain). Human semen was obtained by masturbation and collected with posterior liquefaction in sterile plastic containers from volunteer’s healthy young men. Samples were processed for protein extraction as has been previously reported [32] with modifications.

#### Detection of DAT, NET and SERT monoamine transporters

With the aim of detecting the presence of the dopamine transporter in sperm, as well as the norepinefrine and serotonin transporters, indirect immunofluorescence and Western blot techniques were used.

#### Indirect immunofluorescence

For immunodetection by indirect fluorescence, bovine, equine and human sperm were utilized according to the protocol described by Ramírez et al., (2009) [19], with some modifications. Smears of the different sperm were carried out on new slides, fixed with 4% paraformaldehyde and then blocked for 60 minutes at room temperature (5% BSA, 0.3% Triton x-100 in phosphate buffered saline, pH 7.4). After this, they were incubated overnight with the different primary antibodies specific for DAT, NET, SERT, in a moist chamber at 4°C. As a negative control, each one of the primary antibodies was preabsorbed with the corresponding immunogenic epitope. Later incubation with the secondary antibody was performed, with antibodies being conjugated to fluorochrome Alexa 488 for 60 minutes at room temperature, in a moist chamber, before finally being mounted for observation with an epifluorescence microscope. Negative controls were obtained when sperm samples were incubated with preabsorbed antibodies with the corresponding peptide for DAT (data not shown).

#### Western blot

Samples were processed for protein extract preparation as has been previously reported [32] with modifications. Protein extracts from rat brain, equine, bovine and human sperm were homogenized in extraction buffer (10 mM Tris HCl pH 7.5, 15 mM EDTA, 150 mM NaF, 0.6 M sucrose, 15 mM 2-mercaptoethanol, 1 mM benzamidine, 1 mM sodium orthovanadate, 1 mM PMSF). In brief, samples were frozen at −80°C for 40 minutes in extraction buffer; the extracts were then rapidly thawed and sonicated at 50% power, in two rounds of 10 seconds, each one in ice, frozen again at −80°C for 40 minutes, rapidly thawed once again and were passed ten times through a tuberculin syringe. Samples were quantified using the Bradford method [33]. 80 µg whole protein extract from sperm were separated by SDS-PAGE and electrotransfer to nitrocellulose membrane for immunoblotting using a primary antibodies, diluted 1∶400 in blocking solution (0.3% Tween-20, 1% BSA and 5% skimmed milk) and incubated overnight, continuously stirred, at room temperature. For complementary Western blot analysis to verify the specifity of antibodies, the samples were denaturated at 65°C for 20 min in loading buffer with 6 M Urea For immunodetecction reaction we used primary antibodies blocked with their specific antigens before being incubated with the membranes. The following day, membranes were washed in 0.1% Tween-20 solution in phosphate saline buffer and then incubated with their respective secondary antibodies conjugated to peroxidase. Finally, immunoreactive signal was revealed with a solution of luminol-hydrogen peroxide (ECL, Thermo Sci.) and exposed on photographic film.

#### Incorporation and saturation of ASP^+^ transport

Dopamine transport was performed as described by Mason et al., (2005) [28]. For ASP^+^ incorporation experiments, equine sperm were incubated with 8 µM ASP^+^ for 10 min at 37°C. A total of 240 readings were made, every 5 seconds. Saturation curves were tested at different concentrations of ASP^+^ (0 to 30 µM) at a fixed reading time of 20 min. All ASP^+^ dilutions were performed in PBS with 30 µM Trypan blue to eliminate extracellular fluorescence. Changes in intracellular ASP^+^ concentration were quantified by the increase in fluorescence emitted at 609 nm, readings performed with a microplate reader.

#### Analysis of dopamine effects on sperm physiology

We have previously shown that high dopamine doses inhibit capacitation and motility [19]. In an aim to elucidate whether DAT activity is involved in the high dopamine dose effect, incubation with 0.01, 0.1 and 1 mM dopamine was performed, with and without DAT inhibitors, to discover how this treatment affects sperm viability, motility and acrosomal integrity. The capacitation medium consisted of Whitten’s medium supplemented with BSA (7% final) and sodium bicarbonate (25 mM final).

#### Viability Analysis

This parameter was analyzed with the viability module of the computer-assisted sperm analysis (CASA) system (SCA Microptics), using a biochromatic fluorescent stain based on the use of acridine orange (10 µM) and ethidium bromide (2.5 µM) fluorophores in a protocol modified from Cortés-Gutiérrez et al., (2008) [34]. Smears of each of the times (0, 1, 3 and 6 hours) and treatments (control, 0.01, 0.1 and 1 mM dopamine and 1 mM dopamine plus 10 µM of the DAT inhibitor, GBR12909) were prepared to a dilution ratio of 1∶1 between the samples and a mixture of both fluorochromes. They were then observed with an epifluorescent microscope coupled to the CASA system, with a 10X objective. A minimum of 500 sperms were counted per experiment. Living sperm incorporate the acridine orange, which joins with DNA, emitting a green color on excitation at 488 nm. Dead sperm show damage to the plasma membrane and are permeable for ethidium bromide, which joins with DNA and emits a red colour when excited at 488 nm.

#### Acrosomal integrity

The acrosomal status analysis or acrosomal reaction in sperm after dopamine incubation was performed exactly as described Ramírez et al., (2009) [19], without modifications. The number of intact acrosomes after dopamine treatment was normalized using the number of intact acrosomes at basal conditions before the assay. The results were expressed as a ratio between sperm treated after 1, 3 and 6 hours and non-treated before starting the assay.

#### Total and progressive motility

This parameter was analyzed with the motility module of the CASA system (SCA Microptics). To perform this analysis, 5 µl samples of each one of the treatments were used, mounted onto a slide pre-warmed to 37°C and covered with a coverslip. Samples were analyzed in a microscope coupled to a high-speed camera (takes 25 images per second of various fields), at a magnification of 200X. Images were processed and analyzed with the software, Sperm Class Analyzer 4.2.0.1. Progressive sperm were defined as those with values of straightness coefficient (STR)>75% and with linearity coefficient (LIN)>50%. Spermatozoa were also classified as slow, medium and fast following their curvilinear velocity (VCL) values. In this classification, slow sperm were those with VCL between 10 µm/sec and 45 µm/sec, medium sperm had a VCL between 46 µm/sec and 90 µm/sec and fast sperm were those with VCL>90 µm/sec. Spermatozoa with VCL values below 10 µm/sec were considered non motile.

#### Statistical analysis

For time-dependent ASP^+^ incorporation assays, saturation curves, inhibition of ASP^+^ transport, the effect of dopamine on viability, acrosomal reaction, total and progressive motility and DAT inhibition, the mean ± the standard error (SEM) of the medium was displayed graphically. The different assays were analyzed statistically with a Student’s *t*-test with Bonferroni correction for viability and variance analysis (ANOVA), using GraphPad v5.0 for Windows software. A difference was considered to be statistically significant and highly significant when p<0.05 and p<0.01 respectively.

## Supporting Information

Figure S1 **Presence of Dopamine Transporter (DAT) in mammalian sperm.** Eigthteen micrograms of total protein extract from boar (line 1), human (line 2), stallion (line 3), and bull (line 4) sperm were analyzed by SDS-PAGE and Western blot using a specific human anti-DAT antibody. Positions of relevant molecular weight standards are indicated on the left. Images are representative of three independent experiments.
(TIF)
