# Supplementary figures and images for: Presence and Function of Dopamine Transporter (DAT) in Stallion Sperm: Dopamine Modulates Sperm Motility and Acrosomal Integrity
Source: PLoS One. 2014 Nov 17;9(11):e112834. doi: 10.1371/journal.pone.0112834 (PMC4234536; doi:10.1371/journal.pone.0112834)

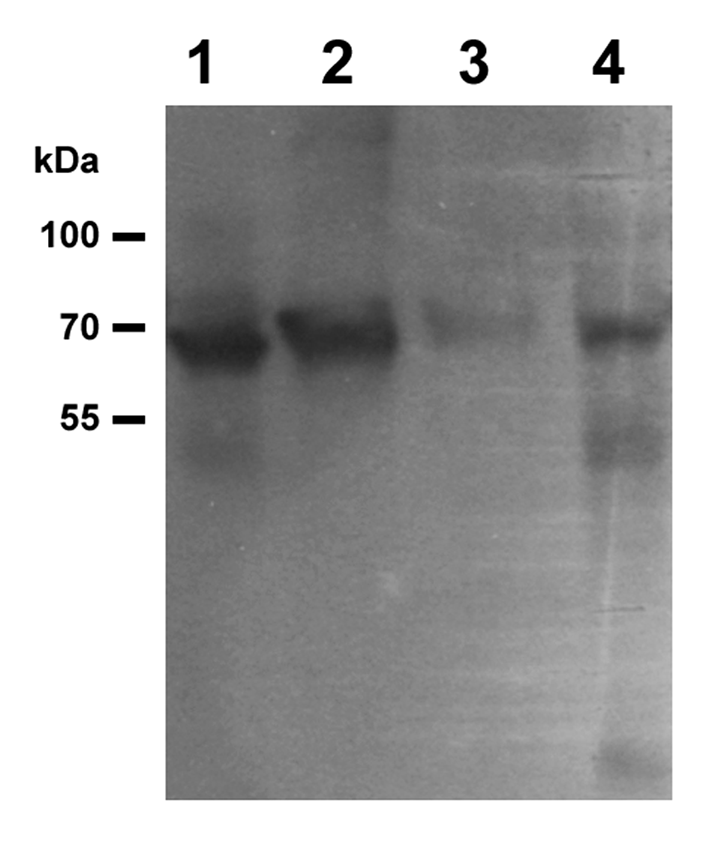

Supplement: Figure S1 — Presence of Dopamine Transporter (DAT) in mammalian sperm. Eigthteen micrograms of total protein extract from boar (line 1), human (line 2), stallion (line 3), and bull (line 4) sperm were analyzed by SDS-PAGE and Western blot using a specific human anti-DAT antibody. Positions of relevant molecular weight standards are indicated on the left. Images are representative of three independent experiments. (TIF) [file pone.0112834.s001.tif]
